# The Influence of Psychological Capital on Innovative Behavior: Examining the Mediating Mechanism of Job Crafting in Operating Room Nurses

**DOI:** 10.1155/jonm/6177341

**Published:** 2026-05-06

**Authors:** Xiao Liang, Zhijiang Li, Wei Liang, Dan Lu, Yu Tang, Chaosong Wu, Junhui Cui

**Affiliations:** ^1^ Department of Operating Room, The Affiliated Jiangning Hospital of Nanjing Medical University, Nanjing, 211100, Jiangsu, China, njmu.edu.cn; ^2^ School of Nursing, Fudan University, Xuhui District, Shanghai, 200032, China, fudan.edu.cn; ^3^ School of Nursing, Nanjing Medical University, Nanjing, 211166, Jiangsu, China, njmu.edu.cn

**Keywords:** innovative behavior, job crafting, operating room nurses, psychological capital

## Abstract

**Aim:**

This study aimed to assess the levels of psychological capital, job crafting, and innovative behavior among operating room nurses and to examine the mediating mechanism of job crafting in the relationship between psychological capital and innovative behavior.

**Methods:**

A multicenter cross‐sectional study was conducted. In June 2025, a total of 361 operating room nurses from six tertiary hospitals in Nanjing, China, were recruited via convenience sampling. Data were collected electronically using validated scales: Demographic Questionnaire, the Psychological Capital Questionnaire, the Job Crafting Scale, and the Nurse Innovative Behavior Scale. Data were analyzed using SPSS 26.0 and AMOS 28.0. Structural equation modeling with bootstrapping tested the mediation model, controlling for educational level and specialist nurse status.

**Results:**

Participants reported moderate‐to‐high levels of psychological capital, job crafting, and innovative behavior. Correlation analysis revealed that innovative behavior was significantly and positively correlated with both psychological capital and job crafting. Hierarchical regression analysis, controlling for significant covariates, showed that both psychological capital and job crafting contributed significantly to the variance in innovative behavior. Mediation analysis demonstrated that job crafting partially mediated the relationship between psychological capital and innovative behavior.

**Conclusions:**

Operating room nurses reported moderately high levels of psychological capital, job crafting, and innovative behavior. Furthermore, job crafting was identified as a significant partial mediator in the relationship between psychological capital and innovative behavior.

**Implications for Nursing Management:**

Nursing management can foster innovation in operating room nurses by developing psychological capital and job crafting skills through targeted training and academic support.

## 1. Introduction

Innovation has long been recognized as a central driving force behind the advancement of nursing as a discipline and the continuous improvement of clinical practice [1]. According to the International Council of Nurses [2], innovation in the nursing field is primarily aimed at addressing evolving patient needs, with a strong emphasis on improving outcomes and care quality through process optimization and technological integration. However, amid rapid advancements in medical technology and the increasing complexity of surgical procedures, operating room nurses are exposed to a high‐pressure, high‐stakes work environment characterized by fast‐paced routines, strict procedural standards, and an expectation of zero errors. In such conditions, nurses’ capacity for innovation becomes a critical factor in maintaining high‐quality healthcare delivery [3–5]. Although numerous studies indicate that nurses frequently generate innovative ideas in clinical settings, many of these ideas fail to materialize due to limited resources, time constraints, and insufficient institutional support [6]. Furthermore, a qualitative study conducted in China revealed that more than half of the operating room nurses experienced reduced motivation to innovate due to burnout and emotional exhaustion [7–9]. Therefore, a deeper understanding of both individual and organizational factors influencing innovation among operating room nurses—particularly from the perspective of psychological capital and its potential role in enabling work‐related behavioral changes—is essential for fostering a supportive environment and enhancing the innovative capacity of nursing teams.

Innovative behavior refers to the proactive actions taken by nurses to address clinical challenges and improve patient care through the initiation, adoption, and implementation of new methods, techniques, or approaches [10]. Within the context of operating room nursing, such behaviors may range from minor but meaningful improvements in workflow—such as optimizing the arrangement of surgical instruments to enhance efficiency—to the creative resolution of unexpected intraoperative situations, thereby improving patient safety and team coordination [6, 11, 12]. Research has consistently shown that while organizational support and a positive team climate are essential for fostering innovation, individual psychological states and intrinsic motivation also play a crucial role in shaping innovative behaviors [13, 14]. Therefore, examining the influence of individual psychological capital on the innovative behaviors of operating room nurses is not only valuable for understanding the underlying psychological mechanisms but also offers new insights for developing interventions aimed at enhancing nurses’ innovative capacity.

Psychological capital refers to a positive psychological resource that individuals develop through their experiences in work and life, contributing to personal growth and resilience [15]. Evidence suggests that psychological capital serves as a key resource for coping with stress and adversity, and plays a vital role in motivating proactive behaviors [16]. A large‐scale study by Yan et al. [17] involving 4677 Chinese nurses found a significant positive association between psychological capital and the likelihood of engaging in innovative practices. In essence, nurses with higher levels of psychological capital are more likely to view challenges as surmountable, exhibit greater emotional regulation, and maintain a goal‐oriented mindset, making them more open to experimenting with new approaches and taking calculated risks in their professional practice [18]. Therefore, psychological capital may facilitate innovation by encouraging nurses to reshape their work experiences in meaningful ways.

Job crafting can be defined as the process through which nurses proactively adjust their tasks, role boundaries, and interpersonal interactions to better align with their work environment [19]. In high‐intensity, high‐stress settings such as the operating room, nurses often reshape their roles by modifying task structures, expanding responsibilities, or redefining the meaning of their work to cope with dynamic professional demands [20]. This concept is grounded in the Job Demands–Resources (JD‐R) theory, which posits that personal and organizational resources—such as psychological capital—can promote proactive behaviors like job crafting, ultimately enhancing role performance and innovation [21]. Compared to individuals with limited resources, those who possess greater personal resources are more inclined to reshape their work experiences to enhance a sense of control and purpose, thereby creating opportunities for innovative actions. Empirical studies have shown that job crafting positively predicts nurses’ innovative behaviors [22], largely due to its ability to strengthen a sense of agency and initiative in the workplace. Moreover, psychological capital has been identified as a critical personal resource that significantly promotes job crafting [23, 24]. Nurses with high psychological capital are more confident in facing challenges and more willing to explore alternative ways of working, which increases their likelihood of engaging in proactive job crafting and contributing to both personal and organizational innovation.

A review of existing literature indicates a strong interconnection between psychological capital, job crafting, and innovative behavior among nursing professionals. However, recent systematic reviews and empirical studies highlight that research specifically focused on operating room nurses remains limited, particularly regarding the actual levels and interrelationships of these variables [25, 26]. Furthermore, the mechanisms through which psychological capital influences innovative behavior are not yet fully understood, and the potential mediating mechanism of job crafting in this relationship lacks empirical validation. To address these gaps, this study aims to investigate the current status of psychological capital, job crafting, and innovative behavior among operating room nurses, and to explore the underlying mechanisms through which psychological capital influences innovative behavior, highlighting the mediating role of job crafting.

**FIGURE 1:**
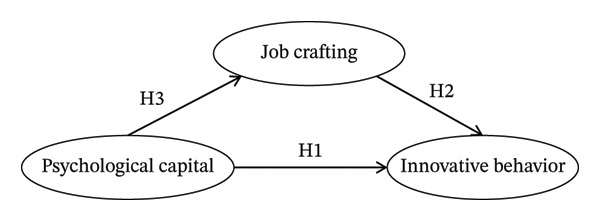
Hypothetical model. Note: H1 = Hypothesis 1; H2 = Hypothesis 2; H3 = Hypothesis 3.

In summary, the JD‐R theory provides a robust framework for understanding these interrelationships. According to the JD‐R model, personal resources (such as psychological capital) can directly buffer job stress and enhance positive organizational outcomes, leading to our first premise that psychological capital promotes innovative behavior (Hypothesis 1) [17]. Furthermore, the theory posits that proactive behaviors, such as job crafting, act as a catalyst for improving work performance and innovation (Hypothesis 2) [22]. Crucially, the JD‐R theory suggests a sequential motivational process where personal resources stimulate proactive job crafting, which subsequently drives positive outcomes [21, 23, 24]. Thus, we postulate that job crafting serves as a vital bridge linking psychological capital to innovative behavior (Hypothesis 3). Based on this theoretical and empirical foundation, the following hypotheses are proposed (see Figure 1):

• Hypothesis 1: Psychological capital is directly and positively associated with innovative behavior among operating room nurses.
• Hypothesis 2: Job crafting is directly and positively associated with innovative behavior among operating room nurses.
• Hypothesis 3: Job crafting partially mediates the relationship between psychological capital and innovative behavior among operating room nurses.

## 2. Methods

### 2.1. Study Design

This study adopted a multicenter cross‐sectional design. The reporting of this study follows the guidelines of the Strengthening the Reporting of Observational Studies in Epidemiology (STROBE) statement [27].

### 2.2. Setting and Participants

Data were collected in June 2025 from six tertiary‐level hospitals in Nanjing, China. These hospitals represent the highest level of medical accreditation in the country, equipped with advanced facilities and experienced in managing complex and critical cases. A convenience sampling approach was used to recruit 361 operating room nurses for participation in the survey. Inclusion criteria were as follows: (1) registered nurse with at least one year of experience in operating room clinical work; (2) direct involvement in perioperative care; and (3) voluntary completion of the informed consent form. Exclusion criteria included: (1) temporary staff such as interns or visiting nurses and (2) absence from work for more than 3 months during the data collection period due to leave or training.

According to general recommendations for structural equation modeling (SEM), a minimum sample size of 200 is typically advised [28]. To further determine the required sample size, we used the SEM‐specific calculator on Daniel Soper’s statistical computation platform∗, entering the following parameters: (1) model structure: 3 latent variables with a total of 11 observed indicators; (2) statistical parameters: medium effect size (0.3), statistical power of 0.95, and alpha level of 0.01. The calculated minimum required sample size was 237. Considering an estimated 20% attrition rate, the target sample size was set at 297 participants. This number satisfies two key criteria: (a) ensuring model stability for SEM analysis and (b) meeting statistical power requirements for estimating complex model parameters. ∗Link: https://www.danielsoper.com/statcalc/calculator.aspx?id=89

### 2.3. Data Collection Instruments

The study employed a variety of validated instruments, including a demographic questionnaire, the Job Crafting Scale (JCS), the Psychological Capital Questionnaire–Revised (PCQ‐R), and the Nurse Innovative Behavior Scale (NIBS).

#### 2.3.1. Demographic Questionnaire

A self‐designed sociodemographic questionnaire was used to collect basic sociodemographic and occupation‐related information, including gender, age, educational level, professional title, position, monthly income (in RMB), marital status, employment type, and specialist nurse status.

#### 2.3.2. PCQ‐R

To assess the level of psychological capital among nurses, we used the Chinese version of PCQ‐R, adapted and validated by Luo and He [15], which was originally developed by Luthans et al. [29]. The scale consists of 20 items across four dimensions: efficacy (6 items), hope (6 items), resilience (5 items), and optimism (3 items). Each item is rated on a 6‐point Likert scale ranging from 1 (strongly disagree) to 6 (strongly agree), with total scores ranging from 20 to 120. Higher scores indicate higher levels of psychological capital. The Cronbach’s alpha coefficient for the original Chinese version was 0.840, and in this study, the internal consistency of the full scale reached 0.906, indicating strong reliability.

#### 2.3.3. JCS

We used the Chinese version of JCS, translated and adapted by Liao [19], which was originally developed by Tims et al. [30]. The scale comprises 21 items distributed across four subscales: increasing structural job resources (5 items), decreasing hindering job demands (6 items), increasing social job resources (5 items), and increasing challenging job demands (5 items). Items are rated on a 5‐point Likert scale from 1 (“never”) to 5 (“always”), with total scores ranging from 21 to 105. Higher scores reflect higher levels of job crafting. The overall Cronbach’s alpha for the scale in this study was 0.878, indicating good internal consistency.

#### 2.3.4. NIBS

To evaluate innovative behavior among Chinese nurses, we employed the NIBS, developed by Bao et al. [10]. The scale includes 10 items across three dimensions: idea generation (3 items), support obtaining (4 items), and idea realization (3 items). Each item is rated on a 5‐point Likert scale ranging from 1 (“never”) to 5 (“frequently”), with total scores ranging from 10 to 50. Higher scores indicate higher levels of innovative behavior. The original Cronbach’s alpha was 0.879, and in this study, the internal consistency was 0.845, demonstrating acceptable reliability.

### 2.4. Data Collection

An online survey platform (https://www.wjx.cn, a widely used online questionnaire service in China) was used to design and distribute the electronic questionnaire, which was made accessible via a unique QR code. After obtaining approval from the nursing departments of the six participating hospitals, one head nurse from each hospital was appointed as a project coordinator responsible for implementing data collection. All coordinators received standardized training on the study objectives, participant eligibility, and data collection procedures. The questionnaire system used dual verification via IP address and WeChat account to ensure each participant completed the survey only once. All items were mandatory. Prior to the formal survey, a pilot test with 30 participants (more than 10% of the minimum sample size) was conducted to refine the questionnaire content. A total of 372 questionnaires were collected, and 11 were excluded due to logical inconsistencies or patterned responses. Finally, 361 valid responses were included in the final analysis, yielding an effective response rate of 97.04%.

### 2.5. Data Analysis

Statistical analyses were conducted using SPSS 26.0 and AMOS 28.0. Differences in innovative behavior across sociodemographic variables were examined using one‐way ANOVA or independent samples *t*‐tests. Continuous data that met the assumption of normality were presented as mean ± standard deviation. Pearson correlation analysis was used to explore the relationships among psychological capital, job crafting, and innovative behavior. A multiple linear regression model was then constructed to examine the effects of psychological capital and job crafting on innovative behavior, with multicollinearity checked using variance inflation factor (VIF < 5) [31]. To control for common method bias, Harman’s single‐factor test was conducted. Exploratory factor analysis showed that the highest common factor explained less than 40% of the variance [32], indicating that common method bias was within acceptable limits. Finally, SEM was performed using AMOS 28.0 with maximum likelihood estimation [28]. Bootstrap resampling (5000 iterations) was used to test the mediation effect, and a 95% confidence interval that did not include zero was considered statistically significant [33].

### 2.6. Ethical Considerations

The study protocol was approved by the Ethics Review Committee of Jiangning Hospital Affiliated to Nanjing Medical University (Approval No. 2025‐03‐035‐K01). Prior to data collection, all participants were informed about the study purpose, privacy protection measures, and survey instructions. Informed consent was obtained via electronic signature, and participation was entirely voluntary. To ensure data security, the following measures were implemented: (1) Data Collection: Conducted via a professional online survey platform with end‐to‐end encryption and strict access control. (2) Data Storage: Collected data were stored in password‐protected encrypted Excel files accessible only to the research team. (3) Privacy Protection: All personal identifiers were removed, and the principle of anonymity was strictly followed throughout the study. Additionally, all scales and instruments used in this study were publicly available and authorized versions, and no additional usage permissions were required.

## 3. Results

### 3.1. Descriptive Statistics and Univariate Analyses

Univariate analysis was conducted on the innovative behavior scores of 361 operating room nurses. The results showed no statistically significant differences in total innovative behavior scores across gender, professional title, position, marital status, or employment type (*p* > 0.05). However, four variables—age, educational level, monthly income (in RMB), and specialist nurse status—emerged as significant correlates of innovative behavior scores (*p* < 0.05). Specifically, innovation scores increased with age and educational level. Nurses with higher monthly incomes also demonstrated higher levels of innovation. Furthermore, specialist nurse scored significantly higher in innovative behavior than non‐specialists (*p* < 0.001). See Table 1.

**TABLE 1:** Comparison of innovative behavior scores among operating room nurses with different demographic characteristics.

Variable	*n*	Score (mean ± SD)	*t/F*	*p*
Gender			0.960	0.338
Male	51	33.98 ± 5.59		
Female	310	33.09 ± 6.25		
Age			20.504	< 0.001
< 25	91	29.64 ± 5.38		
25 ∼< 35	157	33.43 ± 4.84		
35 ∼< 45	72	35.13 ± 5.83		
≥ 45	41	36.95 ± 8.63		
Educational level			37.774	< 0.001
Associate degree or below	99	29.65 ± 5.37		
Bachelor’s degree	224	33.92 ± 5.21		
Master’s degree or above	38	38.37 ± 8.05		
Professional title			2.313	0.076
Registered Nurse	117	32.06 ± 6.28		
Senior Clinical Nurse	154	33.51 ± 5.69		
Nurse Supervisor	54	34.09 ± 6.14		
Associate Chief Nurse	36	34.39 ± 7.30		
Position			−1.204	0.230
No leadership role	348	33.14 ± 6.12		
Head nurse	13	35.23 ± 7.16		
Monthly income (RMB)			6.884	0.001
< 5000	174	32.21 ± 6.10		
5000 ∼< 10,000	99	32.26 ± 6.34		
≥ 10,000	88	35.15 ± 5.65		
Marital status			0.522	0.594
Married	284	33.28 ± 6.40		
Single	75	32.85 ± 5.22		
Divorced or other	2	37.00 ± 4.24		
Employment type			−0.885	0.377
Permanent staff with establishment	144	32.86 ± 6.38		
Contract‐based employment	217	33.45 ± 6.01		
Specialist nurse			−7.025	< 0.001
No	323	32.48 ± 5.52		
Yes	28	39.45 ± 7.73		

*Note:* The t and F values represent the statistical results from independent samples *t*‐tests and one‐way ANOVA, respectively. A *p*‐value < 0.05 indicates statistical significance.

Abbreviation: SD, standard deviation.

### 3.2. Variable Scores

This study further examined the overall performance of operating room nurses across psychological capital, job crafting, and innovative behavior. As shown in Table 2, the total psychological capital score was 89.97 ± 15.44, with an average item score of 4.50 ± 0.77. Among the subscales, “efficacy” had the highest mean score (4.58 ± 0.87), while “resilience” scored relatively lower (4.05 ± 0.81).

**TABLE 2:** Psychological capital, job crafting, and innovative behavior scores among operating room nurses.

Items	Number of items (*n*)	Total score (mean ± SD)	Item mean (mean ± SD)
Psychological capital	20	89.97 ± 15.44	4.50 ± 0.77
Efficacy	6	27.50 ± 5.19	4.58 ± 0.87
Hope	6	27.20 ± 4.94	4.53 ± 0.82
Resilience	5	21.58 ± 4.05	4.05 ± 0.81
Optimism	3	13.69 ± 2.94	4.56 ± 0.98
Job crafting	21	65.08 ± 11.88	3.10 ± 0.57
Increasing structural job resources	5	15.65 ± 3.12	3.13 ± 0.62
Decreasing hindering job demands	6	16.96 ± 3.98	2.83 ± 0.66
Increasing social job resources	5	16.29 ± 3.46	3.26 ± 0.69
Increasing challenging job demands	5	16.17 ± 3.26	3.23 ± 0.65
innovative behavior	10	33.21 ± 6.16	3.32 ± 0.62
Idea generation	3	10.39 ± 2.16	3.46 ± 0.72
Support obtaining	4	12.96 ± 2.66	3.24 ± 0.67
Idea realization	3	9.86 ± 2.20	3.29 ± 0.73

The total job crafting score was 65.08 ± 11.88, with an average item score of 3.10 ± 0.57. Among the four subscales, “decreasing hindering job demands” had the lowest score (2.83 ± 0.66), while “increasing social job resources” scored the highest (3.26 ± 0.69).

The total innovative behavior score was 33.21 ± 6.16, with an average item score of 3.32 ± 0.62. Subscale scores were generally balanced, with “idea generation” scoring slightly higher (3.46 ± 0.72).

### 3.3. Pearson Correlation Analysis

Pearson correlation analysis revealed a significant positive correlation between psychological capital and job crafting (*r* = 0.681, *p* < 0.001). A significant positive relationship was also found between psychological capital and innovative behavior (*r* = 0.609, *p* < 0.001). Additionally, job crafting was significantly correlated with innovative behavior (*r* = 0.614, *p* < 0.001). See Table 3.

**TABLE 3:** Correlation among psychological capital, job crafting, and innovative behavior among operating room nurses.

	**1**	**2**	**3**	**4**	**5**	**6**	**7**	**8**	**9**	**10**	**11**	**12**	**13**	**14**

1	1													
2	0.900^∗∗∗^	1												
3	0.921^∗∗∗^	0.776^∗∗∗^	1											
4	0.882^∗∗∗^	0.670^∗∗∗^	0.740^∗∗∗^	1										
5	0.901^∗∗∗^	0.737^∗∗∗^	0.768^∗∗∗^	0.828^∗∗∗^	1									
6	0.681^∗∗∗^	0.612^∗∗∗^	0.619^∗∗∗^	0.615^∗∗∗^	0.608^∗∗∗^	1								
7	0.649^∗∗∗^	0.571^∗∗∗^	0.583^∗∗∗^	0.596^∗∗∗^	0.598^∗∗∗^	0.871^∗∗∗^	1							
8	0.575^∗∗∗^	0.519^∗∗∗^	0.524^∗∗∗^	0.527^∗∗∗^	0.499^∗∗∗^	0.850^∗∗∗^	0.678^∗∗∗^	1						
9	0.551^∗∗∗^	0.497^∗∗∗^	0.500^∗∗∗^	0.500^∗∗∗^	0.486^∗∗∗^	0.846^∗∗∗^	0.653^∗∗∗^	0.564^∗∗∗^	1					
10	0.573^∗∗∗^	0.522^∗∗∗^	0.528^∗∗∗^	0.496^∗∗∗^	0.518^∗∗∗^	0.875^∗∗∗^	0.696^∗∗∗^	0.631^∗∗∗^	0.710^∗∗∗^	1				
11	0.609^∗∗∗^	0.525^∗∗∗^	0.577^∗∗∗^	0.540^∗∗∗^	0.557^∗∗∗^	0.614^∗∗∗^	0.556^∗∗∗^	0.546^∗∗∗^	0.507^∗∗∗^	0.499^∗∗∗^	1			
12	0.530^∗∗∗^	0.478^∗∗∗^	0.496^∗∗∗^	0.446^∗∗∗^	0.493^∗∗∗^	0.560^∗∗∗^	0.496^∗∗∗^	0.449^∗∗∗^	0.495^∗∗∗^	0.492^∗∗∗^	0.828^∗∗∗^	1		
13	0.548^∗∗∗^	0.474^∗∗∗^	0.512^∗∗∗^	0.492^∗∗∗^	0.502^∗∗∗^	0.532^∗∗∗^	0.500^∗∗∗^	0.487^∗∗∗^	0.420^∗∗∗^	0.420^∗∗∗^	0.915^∗∗∗^	0.623^∗∗∗^	1	
14	0.522^∗∗∗^	0.428^∗∗∗^	0.510^∗∗∗^	0.480^∗∗∗^	0.468^∗∗∗^	0.526^∗∗∗^	0.466^∗∗∗^	0.501^∗∗∗^	0.428^∗∗∗^	0.407^∗∗∗^	0.882^∗∗∗^	0.584^∗∗∗^	0.742^∗∗∗^	1

*Note:* 1. Psychological capital; 2. Efficacy; 3. Hope; 4. Resilience; 5. Optimism; 6. Job crafting; 7. Increasing structural job resources; 8. Decreasing hindering job demands; 9. Increasing social job resources; 10. Increasing challenging job demands; 11. Innovative behavior; 12. Idea Generation; 13. Support obtaining; 14. Idea realization.

^∗∗∗^ *p* < 0.001.

### 3.4. Measurement Model and Validity

Prior to hypothesis testing, the normality of the data was assessed. The skewness and kurtosis values for all observed variables fell within the acceptable range (skewness < |3|, kurtosis < |10|), indicating a normal distribution suitable for SEM. A confirmatory factor analysis (CFA) was conducted to assess the measurement model. The results indicated an excellent fit: *χ* ^2^/df = 2.779, RMSEA = 0.070, CFI = 0.965, TLI = 0.955. Furthermore, convergent validity was established as the composite reliability (CR) for psychological capital, job crafting, and innovative behavior was 0.925, 0.884, and 0.841, respectively, all exceeding the recommended threshold of 0.70. The average variance extracted (AVE) values were 0.755, 0.657, and 0.639, respectively, all exceeding the 0.50 threshold. Finally, discriminant validity was confirmed, as the square root of the AVE for each construct (0.869, 0.811, and 0.799, respectively) was greater than their inter‐construct correlations.

### 3.5. Hierarchical Regression Analysis

The VIF for all variables was below 5, indicating no serious multicollinearity issues in the model. A hierarchical regression analysis was performed using total innovative behavior as the dependent variable. In the first step, demographic variables that showed significance in univariate analysis—age, educational level, monthly income, and specialist nurse—were included, explaining 21.6% of the variance. In the second step, psychological capital was added, increasing the explained variance to 46.1%. Finally, job crafting was introduced in the third step, raising the total explained variance to 52.9%. These results suggest that both psychological capital and job crafting are significant predictors of innovative behavior among operating room nurses. See Table 4.

**TABLE 4:** Hierarchical regression analysis of innovative behavior among operating room nurses (*n* = 361).

Variables	Model 1					Model 2					Model 3				
	*B*	*SE*	*β*	*t*	*p*	*B*	*SE*	*β*	*t*	*P*	*B*	*SE*	*β*	*t*	*p*
Constant	21.469	1.279		16.792	< 0.001	6.741	1.568		4.298	< 0.001	3.635	1.526		2.382	0.018
Age	0.689	0.475	0.105	1.452	0.147	0.631	0.394	0.096	1.603	0.110	0.715	0.368	0.109	1.943	0.053
Educational level	2.475	0.794	0.238	3.118	0.002	1.372	0.664	0.132	2.066	0.040	1.267	0.620	0.122	2.042	0.042
Monthly income	1.307	0.352	0.174	3.715	< 0.001	0.358	0.301	0.048	1.191	0.235	0.284	0.281	0.038	1.011	0.313
Specialist nurse	3.087	1.165	0.154	2.650	0.008	2.828	0.966	0.141	2.926	0.004	2.603	0.903	0.130	2.882	0.004
Psychological capital						0.209	0.016	0.525	12.745	< 0.001	0.113	0.020	0.285	5.609	< 0.001
Job crafting											0.186	0.026	0.359	7.268	< 0.001
*R* ^2^	0.225					0.468					0.537				
Adjusted *R* ^2^	0.216					0.461					0.529				
*F*	25.825					62.518					68.508				
*P*	< 0.001					< 0.001					< 0.001				

*Note:* Variable coding: Age: 1 = < 25, 2 = 25 ∼< 35, 3 = 35 ∼< 45, 4 = ≥ 45; Educational level: 1 = Associate degree or below, 2 = Bachelor’s degree, 3 = Master’s degree or above; Monthly income: 1 = < 5000, 2 = 5000 ∼< 10,000, 3 = ≥ 10,000; Specialist nurse: 1 = No, 2 = Yes; Psychological Capital and Job Crafting entered as raw scores. B = Unstandardized coefficient; *β* = Standardized coefficient; *R* ^2^ = Coefficient of determination.

Abbreviation: SE, Standard Error.

### 3.6. Mediation Analysis

The Harman single‐factor test extracted 9 initial factors with eigenvalues greater than 1. The first factor explained only 32.481% of the total variance, indicating that common method bias was not a serious issue and measurement validity was acceptable.

**FIGURE 2:**
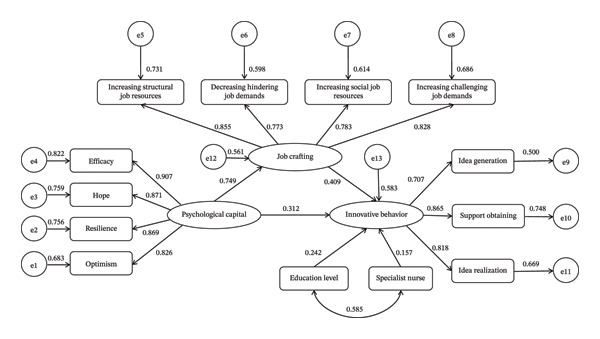
The association between psychological capital and innovative behavior: examining the mediating role of job crafting in operating room nurses (standardized version).

In the SEM, psychological capital was set as the independent variable, job crafting as the mediator, and innovative behavior as the outcome. Educational level and specialist nurse were included as covariates (see Figure 2). Maximum likelihood estimation was used for parameter estimation. As shown in Table 5, all fit indices met acceptable standards, indicating good model‐data fit.

**TABLE 5:** Model fit indices.

Fit index	Criteria	Model result	Interpretation
*χ* ^2^/df	< 3 (excellent), < 5 (acceptable)	2.779	Excellent
RMSEA	< 0.05 (excellent), < 0.08 (acceptable)	0.070	Acceptable
CFI	> 0.95 (excellent), > 0.90 (acceptable)	0.965	Excellent
TLI	> 0.95 (excellent), > 0.90 (acceptable)	0.955	Excellent
GFI	> 0.90 (excellent), > 0.80 (acceptable)	0.932	Excellent
NFI	> 0.90 (excellent), > 0.80 (acceptable)	0.946	Excellent
RFI	> 0.90 (excellent), > 0.80 (acceptable)	0.932	Excellent
IFI	> 0.90 (excellent), > 0.80 (acceptable)	0.965	Excellent

Bootstrap analysis indicated a significant direct association between psychological capital and innovative behavior, with a standardized estimate of 0.312 (95% CI: 0.259 ∼ 0.365), accounting for 50.49% of the total association. Additionally, job crafting partially mediated the relationship between psychological capital and innovative behavior, with an estimated indirect path value of 0.306 (95% CI: 0.267∼0.345). Job crafting thus explained 49.51% of the overall relationship between psychological capital and innovative behavior. See Table 6 for detailed parameter estimates.

**TABLE 6:** The association between psychological capital and innovative behavior: examining the mediating role of job crafting in operating room nurses (standardized).

Path	β	SE	95% CI		p	Proportion (%)
			Lower	Upper		
Direct path	0.312	0.027	0.259	0.365	< 0.001	50.49
Indirect path	0.306	0.020	0.267	0.345	< 0.001	49.51
Total path	0.618	0.024	0.571	0.665	< 0.001	100.00

*Note: β* = Standardized path coefficient. The indirect path represents the mediating role of job crafting.

Abbreviations: CI, confidence interval; SE, standard error.

## 4. Discussion

Psychological capital and job crafting were found to be significantly and positively associated with innovative behavior among operating room nurses. SEM revealed that job crafting partially mediated the relationship between psychological capital and innovative behavior, accounting for 49.51% of the total effect.

The present study showed a generally moderate to high level of job crafting among operating room nurses. This finding is consistent with recent studies by Zhang et al. [13] and Liang et al. [34]. The relatively high level of job crafting may be attributed to the high‐pressure, high‐risk, and fast‐paced nature of the operating room environment, which drives nurses to proactively adapt and optimize their workflows, thereby developing strong problem‐solving skills and a proactive mindset [3, 35]. Among the subscales, “increasing social job resources” scored the highest, whereas “decreasing hindering job demands” received the lowest scores. The former reflects the highly collaborative nature of the operating room and the nurses’ natural tendency to seek social support, enabling them to build stronger relationships with colleagues and physicians, gain guidance or emotional support, and enhance information sharing, skill development, and psychological resilience [36]. However, compared to actively acquiring resources, nurses appeared to be less proficient in identifying and reducing unreasonable job demands. This may limit their ability to optimize processes, refuse non‐core tasks, or negotiate reasonable workloads. This limitation may stem not only from individual differences in strategy application (e.g., task reorganization, boundary setting) but also from the lack of organizational empowerment and institutional support [37]. Therefore, enhancing nurses’ autonomy in decision‐making and improving institutional support systems may be key strategies to strengthen performance in this area.

Our findings indicated that the overall level among operating room nurses was above average. While this result was lower than that reported by Mi et al. [38], it was higher than the findings of Zhu et al. [39]. Among the four dimensions, efficacy scored the highest, while resilience scored the lowest. These differences may be attributed to the fact that operating room nurses, due to their frequent exposure to critical and emergency situations, tend to develop strong self‐efficacy over time. However, the high‐intensity workload, limited recovery time, and lack of systematic psychological support may compromise their resilience when facing persistent stress [40, 41]. Therefore, nursing managers are encouraged to implement targeted resilience training programs, optimize shift scheduling to reduce physical and mental strain, and provide accessible psychological counseling services to promote mental well‐being and ensure the delivery of high‐quality patient care.

The overall innovative behavior score among operating room nurses was found to be moderately high, surpassing the findings reported by Jiang et al. [42], although slightly lower than those observed in studies by Miao et al. [35] and Wang et al. [43]. Among the subscales, “idea generation” scored the highest, whereas “support obtaining” was the weakest. These differences may be attributed to the unique characteristics of the operating room environment. On one hand, the fast‐paced and high‐pressure nature of the setting encourages nurses to accumulate practical experience and develop strong capabilities in identifying problems and proposing improvements [44]. On the other hand, the lack of organizational mechanisms to encourage innovation and the low adoption rate of nurse‐initiated suggestions, coupled with limited interdepartmental collaboration, may hinder the actual implementation of these ideas, thereby affecting the “support obtaining” dimension [45]. Therefore, nursing leaders should establish systematic innovation support systems, such as innovation funding programs, interdisciplinary collaboration platforms, and regular innovation training sessions, to ensure that nurses’ innovative potential can be effectively translated into practice.

The study found that psychological capital had a significant positive effect on innovative behavior among operating room nurses, consistent with findings by Zhang et al. [11] in nursing populations, supporting Hypothesis 1. Furthermore, job crafting was also significantly associated with innovative behavior, aligning with the findings of Wu et al. [22], confirming Hypothesis 2. These results confirm that personal resources and proactive work adjustments collaboratively enhance nurses’ sense of autonomy, thereby stimulating intrinsic motivation for innovation [46].

The mediation analysis revealed that job crafting partially mediated the relationship between psychological capital and innovative behavior, explaining 49.51% of the total effect, thus supporting Hypothesis 3. This finding not only deepens our understanding of the mechanisms through which psychological capital influences innovation but also provides empirical support for the JD‐R theory within the high‐stress operating room context [21]. It demonstrates that psychological capital, as a vital personal resource, enables nurses to effectively utilize job crafting to manage complex environments, thereby creating the necessary cognitive and behavioral space for innovation [47]. In addition, regression analysis showed that both educational level and specialist nurse status were consistently significant predictors across all three models. When these variables were included in the structural equation model, their significance in relation to innovative behavior was further substantiated. Nurses with higher education levels tend to possess stronger learning abilities, critical thinking, and problem‐solving skills, which enable them to identify opportunities for improvement and propose innovative solutions in complex environments. Similarly, specialist nurses, due to their advanced training and accumulated clinical experience, demonstrate greater autonomy, professional judgment, and practical innovation capabilities, which contribute to continuous quality improvement and innovation in nursing practice.

## 5. Limitations

The cross‐sectional design precludes causal interpretations regarding the relationships among psychological capital, job crafting, and innovative behavior. Second, the use of convenience sampling and the restriction of our sample to operating room nurses exclusively from tertiary hospitals may introduce selection bias. Given that tertiary hospitals typically possess more abundant resources and organizational support, these findings may not be fully generalizable to nurses working in primary or secondary healthcare settings. Furthermore, the exclusive use of quantitative methods may overlook nuanced subjective experiences and contextual factors influencing innovative behavior. Future studies would benefit from longitudinal or mixed‐methods approaches that incorporate qualitative components—such as in‐depth interviews or focus groups—to provide richer insights into motivational processes and contextual barriers. Additionally, including other potential variables such as organizational culture, leadership behaviors, or personality traits could further refine the model.

## 6. Conclusions

This study evaluated the current status of psychological capital, job crafting, and innovative behavior among operating room nurses in tertiary hospitals, revealing generally moderate to high levels across all three domains. Notably, while nurses demonstrated particular strengths in self‐efficacy and seeking social resources, the findings highlighted specific areas requiring intervention, such as resilience and the ability to decrease hindering job demands. Crucially, the results confirmed that both psychological capital and job crafting are positively associated with innovative behavior. Furthermore, job crafting was identified as a vital partial mediator in this relationship, suggesting that internal psychological resources facilitate innovation largely by empowering nurses to proactively redesign and optimize their work tasks. These findings underscore that to cultivate an innovative clinical environment, healthcare organizations must move beyond traditional top‐down training; they should actively nurture nurses’ psychological capital and grant them the autonomy to engage in job crafting, thereby transforming everyday clinical challenges into opportunities for continuous quality improvement.

## 7. Implications for Nursing Management

While this observational study did not directly test the effectiveness of specific interventions, the findings provide a sound basis for several practical recommendations. To systematically foster innovative behavior among operating room nurses, nursing management could prioritize strengthening psychological capital and job crafting capabilities. First, as potential strategies based on our results, implementing psychological capital development programs—such as resilience training, positive leadership workshops, and structured stress management sessions—can help enhance nurses’ self‐efficacy and emotional resilience, establishing a foundation for sustained innovative thinking and problem‐solving. Second, fostering job crafting skills enables nurses to proactively reconfigure tasks, refine communication dynamics, and mobilize social support, thereby cultivating more adaptable and initiative‐driven professional practices. Moreover, considering the pronounced association between higher education, specialist qualifications, and innovative output, nursing leaders should emphasize continuing academic development and specialist training pathways. Advancing specialized education and credentialing will substantially elevate collective expertise and innovative capacity across nursing teams.

## Author Contributions

Xiao Liang: conceptualization, investigation, and writing–original draft. Zhijiang Li: methodology, formal analysis, and writing–original draft. Wei Liang: data curation, formal analysis, and writing–review and editing. Dan Lu: investigation and data curation. Yu Tang: investigation and resources. Chaosong Wu: investigation and visualization. Junhui Cui: conceptualization, supervision, writing–review and editing, and project administration. Xiao Liang, Zhijiang Li, and Wei Liang contributed equally to this work.

## Funding

This research received no specific grant from any funding agency in the public, commercial, or not‐for‐profit sectors.

## Ethics Statement

This study was approved by the Ethics Review Committee of Jiangning Hospital Affiliated to Nanjing Medical University (Approval No.: 2025‐03‐035‐K01). All participants were informed of the study purpose, privacy protection measures, and questionnaire instructions before taking part. Informed consent was obtained via electronic signature, and participation was voluntary. Data were collected through a secure online platform with end‐to‐end encryption and access controls. After download, they were stored in encrypted Excel files accessible only to authorized researchers. Personal identifiers were removed to ensure anonymity. All scales used were publicly available and required no additional permission.

## Conflicts of Interest

The authors declare no conflicts of interest.

## Data Availability

The data that support the findings of this study are available from the corresponding author upon reasonable request.
